# What’s in it for the animals? Symbiotically considering ‘therapeutic’ human-animal relations within spaces and practices of care farming

**DOI:** 10.1136/medhum-2018-011627

**Published:** 2019-08-13

**Authors:** Richard Gorman

**Affiliations:** Geography, University of Exeter College of Life and Environmental Sciences, Exeter EX4 4PY, UK

**Keywords:** human-animal relations, care farming, symbiosis, animal-assisted-therapy, parasitism, mutualism

## Abstract

Human-animal relations are increasingly imbricated, encountered and experienced in the production of medicine and health. Drawing on an empirical study of care farms in the UK, this article uses the language of symbiosis to develop a framework for critically considering the relationships enrolled within interspecies therapeutic practices. Care farming is an emerging paradigm that aims to deploy farming practices as a form of therapeutic intervention, with human-animal relations framed as providing important opportunities for human health. This article moves to attend to multispecies therapeutic interventions and relationships from a more-than-human perspective, drawing attention to the often-troubling anthropocentrism in which such practices are framed and performed. Attempting to perform and realise human imaginations of ‘therapeutic’ affects, spaces and relationships can rely on processes that reduce animals’ own opportunities for flourishing. Yet, the therapeutic use of other species does not have to be forever anthropocentric or utilitarian. The article explores whether relations between humans and animals might result in a level of mutual proliferation of affective capacities, reciprocally beneficial. These human-animal entanglements highlight opportunities to think more critically about how to practice interspecies relationships and practices in ways that are less parasitic, and instead framed more by attempts at producing opportunities for mutualistic flourishing.

## Introduction

‘Why look at animals?’ John Berger famously questioned.1 It remains a productive and provocative question, and one with resonance for the medical humanities. As Atkinson *et al* have argued, contemporary medical humanities research has a key role to play in opening out and interrogating ‘the multiple ways in which “the medical”, medicine and health are encountered and experienced’ (p.73).2 Increasingly, non-human life is being recognised as consequential for human health, used and commodified in attempts to produce affective and healthful encounters.3–5 For scholars in the medical humanities concerned with, what Viney *et al* call, ‘the complex making and re-making of medicine and health’ (p.7),6 the increasing involvement and enrolment of animals into practices associated with health and well-being offers new sites through which to think about the processes and actants imbricated in being (and becoming) well.7

This paper draws on the language of ‘symbiosis’ to develop a framework for more critically exploring the relationships at play within multispecies therapeutic interventions, through an empirical study of care farms in the UK. Care farming is an emerging paradigm that aims to utilise farms, and farming practices, as a form of therapeutic intervention. In this practice, non-human life and multispecies relationships are enrolled in attempts to open up therapeutic possibilities, with human-animal relations framed as providing important opportunities that can come to positively affect an individual’s capacities to function and flourish.8 9

However, recognising that discussions of ‘therapeutic’ relationships with animals mainly focus solely on human considerations of health,10 in this paper I am keen to consider these interactions and relationships from a more-than-human perspective,11 and draw attention to the often-troubling anthropocentrism in which multispecies therapeutic practices are framed and performed. I highlight how certain ‘therapeutic’ relationships that may produce new bodily capacities for humans can emerge through ‘parasitic’ practices. Attempting to perform and realise human imaginaries of ‘therapeutic’ affects, spaces and relationships can rely on processes that interrupt and disrupt animals’ own health capacities and assemblages.

It is also worth recognising that there are certain ethicopolitical stances that view any use of animals to aid in human well-being as an(other) exploitation, regardless of the framing.12 13 My interest here is to consider the emplaced and emergent relationships taking place between humans and animals, rather than attending to the wider structures and ethics of 'carnophallogocentrism' that enable and (re)produce the ‘use’ of animals in such a way.14 Instead, following Haraway, I find myself more intrigued by ‘more modest possibilities of partial recuperation and getting on together’ (p10).15 However, while animal-assisted-therapy has often been critiqued from the standpoint of certain ethical perspectives, care farming has yet to be fully ethically examined to the same extent. This is despite the fact that care farming, at least, in its conventional practice, raises additional moral complexities surrounding our use and relationships with non-human animals. Evans and Grey, writing about the practice and ethics of animal-assisted therapies ask, is it enough that we don’t eat our co-workers?16 Such a question raises challenging provocations for embedding therapeutic interactions with animals in a productive agricultural setting, and I hope that the considerations in this article prove to be a stimulus for more discussions and critical perspectives on using non-human animals for therapeutic purposes.

However, perhaps the therapeutic use of other species does not have to be parasitically anthropocentric or utilitarian. I demonstrate how alternatively, some human-animal relations may be conceptualised as ‘commensal’. These commensal relations emerge when therapeutic benefits for humans are generated in modalities that do little to obtrude on the animals involved—though equally, these commensal interactions do little to advance or assist animals’ own capacities, non-humans becoming positioned more as tools to provoke some form of therapeutic encounter for humans.

While there is a danger of elevating the human experience and relegating non-humans to a state of ‘therapeutic utility’, simply assuming that animals do not receive anything of benefit from these relationships at all is equally problematic. Thus, I additionally move to explore, with cautious optimism, the complexity of whether spaces of care farming can be ‘mutually therapeutic’, considering if, in certain ways and framings, relations between humans and animals within care farming practices and spaces might potentially result in a mutual proliferation of affective capacities, reciprocally beneficial—humans and animals becoming therapeutic together.

## CONTEXTUALISING care farming

Defined by Hine *et al* as ‘the use of commercial farms and agricultural landscapes as a base for promoting mental and physical health through normal farming activity’ (p.247),17 care farming involves inviting various groups into an agricultural space as part of a programme of care, therapy or rehabilitation. In the UK, over 250 farms operate this way, with an estimated 8750 people attending these farms for therapeutic opportunities each week.18

Care farming has been used as an intervention for a wide variety of different groups and conditions, including (but not limited to) autism,19 acquired brain injury,20 dementia,21 people dealing with substance addiction,17 22 people experiencing mental ill-health,23 24 and people within the criminal justice system.25 This frequently occurs in multiuse groups,26 and the idea of ‘mixed populations’ on the farms is often encouraged, a means of drawing people together around shared encounters and experiences, regardless of their individual ‘client group’, background or abilities.3 The types of activities conducted on care farms can vary greatly, visitors to the farms (‘service users’ or ‘co-farmers’) get involved in caring for farm animals (feeding, grooming, mucking out), maintenance work (building and repairing farm structures), ‘nature’ activities (wildlife encounters and conservation work), gardening and horticulture (growing and nurturing plants), as well as other site-specific, project-specific and client-specific activities, all within the specific place-based context of an agricultural landscape.26 Organisationally, most care farms are either commercial farm businesses, charities or community interest companies.18

The farms aim to deliver structured programmes of farming-related and goal-based activities on a regular basis, via day care, supported workplaces and residential places.23 Research on this emerging form of therapeutic intervention and agricultural paradigm is growing, and participation in a care farming programme has been shown to decrease anxiety22 and depressive symptoms24 while reportedly increasing self-esteem,17 social interaction24 and psychological well-being.27

But what is ‘care’ in the context of ‘care farming’? Care is a complicated concept, structured and practised in different ways. It can generally be understood as a provision of practical or emotional support realised through a complex network of actants and actions with multidirectional flows of activity and connexions.28 To draw on Fox, care is a relation which ‘may supply its recipient with new capacities to ‘become-other’ and thereby resist the constraints of illness, disability or ageing’ (p.505).29 Though equally, care relationships have the potential to become constraining as well as enabling.30 ‘Caring for’ is not necessarily rewarding and comforting.31 Importantly, care is a relation which affects the recipient, and transforms, enables and constrains the caregiver too.28

Despite the growing body of academic work on care farming, the multispecies relationships that unfold within the spaces and practices of care farming are often neglected and, as argued by Hassink *et al*, ‘requires further study’ (p.3).9 Non-humans are lively and dynamic colleagues in the making of worlds,32 an important part of an engagement with health and place, not just constituent parts of a homogeneous ‘landscape’.3 Visitors to care farms have the opportunity to become involved in a range of tasks that bring them into direct material, bodily and sensorial encounters with the range of non-human life on the farms. These encounters, interactions and relationships with animals become a catalyst for producing new ways of being with the world, facilitating and stimulating new emotions, knowledges, experiences and socialities.8 Being given a level of responsibility for the care of animals allows people to feel they are doing ‘important’ and ‘useful’ work,9 reframing identities and initiating empowering changes in self-confidence and self-image as participants move to become caregivers, rather than just recipients of care.3 The human-animal relationships and encounters (and here, I follow Hayward that, ‘by ‘encounter’, I mean not necessarily contact or direct meeting but rather a sensuous rapport or energetic cadence’ (p.165)33) that develop within care farms become an important pathway that enables a level of flourishing and well-being.8 34

While scholarship on care farming has begun to think critically about how non-human presence and agency might co-produce ‘therapeutic affects’,9 34 35 recognising the multispecies nature of the spaces and practices of care farming also leads to thinking about animals’ contested positions within these practices,3 and questioning how being part of these ‘therapeutic spaces’ might impact individual animals themselves. There is a danger of elevating the human experience, relegating non-humans to a state of utility. Animals are subjects in their own right. To quote Hayward, ‘as we look for multispecies manifestations we must not ignore the repercussions that these unions have for all actors’ (p.592).36

Thus, within the rest of the article, I move to consider the often-troubling anthropocentrism of the ways in which interspecies therapeutic practices are framed and performed within care farms, as well as questioning, with cautious optimism, whether animals may benefit in certain ways from their relationships with humans within these ‘therapeutic’ spaces. To do so, I draw on the language of symbiosis to explore how parasitic, commensal and mutualistic relationships emerge and are bound up within the performance and imaginaries of multispecies modes of health and well-being.

## CONCEPTUALISING symbiosis

‘Symbiosis may be the greatest enigma in the history of biological terminology’ suggest Martin and Schwab (p.7),37 tracing the ‘chaotic’ confusion and complexity of defining the term. Anton de Bary, a plant pathologist who coined the phrase in 1879, defined it as the ‘living together of dissimilarly named organisms’.37 38

There are many diverse phenomena regarding the living together of organisms of different species that are associated with parasitism, mutualism, etc. They are too diverse and complex to be put into categories. Parasitism, mutualism, and lichenism are special cases in this establishment of associations in which the term symbiosis serves as a general description […] but here, again, we cannot define exact boundaries […] the phenomena that we have described as symbiosis are only specific cases among the many relationships that exist between organisms, these are merely a contribution to understanding the entirety of associations between organisms.

(de Bary, 1878, cited in Oulhen *et al*’s 2016 translation, pp.136–138)38

de Bary’s original introduction of symbiosis operates at quite a broad level, even serving to suggest a level of fluidity between categories of interactions. The concept of symbiosis, though originating in biological science, has a generative potential as a useful language and vocabulary for the medical humanities, particularly when it comes to describing and understanding multispecies relationships—significantly this idea of ‘togetherness’. There are opportunities for developing a more ‘relational’39 modality of approaching symbiosis and the ‘associations between organisms’.38 Indeed, in concluding their review of the terminology of symbiosis, Martin and Schwab call for liberating ‘this beautiful term that simply means “living together”’ (p.42).37

As Haraway argues, ‘co-evolution has to be defined more broadly than biologists habitually do’ (p.31).40 For Haraway, co-evolution provides opportunities to embrace a more ‘naturecultural’ way of understanding interspecies interactions, beyond biological reductionism or cultural uniqueness, and a way of beginning to describe how companion species come to be co-constitutive of one another.40 Similar engagements and thinking with symbiosis can be seen in Helmreich’s idea of symbiopolitics, ‘the governance of relations among entangled living things’ (p.15).41 Helmreich draws on Lynn Margulis’s concept of symbiogenesis—‘the arrival of new biological kinds not (only) through descent with modification, but through incorporation and entanglement’.42 Such a politics of entanglement is necessary to understand health and well-being in the Anthropocene. In more recent work, Haraway discusses ideas of ‘sympoiesis’ — making with; health is never made alone. Haraway suggests describing ‘symbiotic assemblages’ of beings ‘in diverse kinds of relationalities and with varying degrees of openness to attachments’ (p.60).15

Importantly, Haraway reminds us that symbiosis is not a synonym for mutually beneficial, but rather there is an ‘array of names needed to designate the heterogeneous webbed patterns and processes of situated and dynamic dilemmas and advantages’ (p.60).15 While it has not yet been a language of particular interest within the medical humanities, human geographers, such as Hinchliffe43 and Bull,44 45 have drawn on the language of symbiosis to point towards the relational character of change embedded within complex multiply affective relationships.32 Hinchliffe, for example, discusses how heterogeneous actants co-produce ‘opportunities and constraints for one another through all manner of relations including co-operation, symbiosis, parasitism, co-habitation, opportunism as well as competition’ (p.25).43

The focus on the co-production of ‘opportunities and constraints’ through these complex symbiotic relationships aligns well with new materialist and vitalist approaches to understanding health and well-being.46 Symbiosis provides a way of, as Greenhough suggests, paying attention to ‘the ways in which dynamic and changing worlds are lived with and performed through the interactions of living and lively beings’ (p.41).47 New relations and associations produce new ‘bodily capacities’ or close down existing ones;48 the continuous modification and transition of a body’s competencies and potential for action.49 Thus following Emmerson, we might say that healthful encounters are those that transform a body's potencies to ‘do different things’ and ‘perform different actions’. (p.11)50 In such a framing, health is processual, not simply a ‘state’ of an ontologically prior body, nor an outcome to be achieved, but dynamically and relationally constituted.51 52

For Hanlon, co-evolution is a way of describing processes that operate ‘in even the most mundane and routine of daily routines, or in the minutest of instances’ (p.144), but come to affect the processual (re)shaping of capacities through intersections, interactions, and interrelations between different bodies and objects.53 Hanlon goes on to argue that health scholarship might widen its account of co-evolution, to recognise the ‘ways in which bodies not only interact, but co-evolve with things (eg, physical infrastructure, technologies) and other beings (ie, not simply other people, but pets, livestock, wildlife, insects and so on)’ (p.144).53

Exploring symbiotic processes of living together creates a way to attend to Andrews’ calls for health scholarship to better recognise ‘the transactional dynamics of living things’ (p.211).46 Symbiosis thus provides a useful analytical framework through which to consider the multiple ways that practices of health and medicine are encountered and experienced; how relations and associations between heterogeneous actants differently define, enable and enact what different actants may become; and highlight the contested and multiple ways that places and practices of health and well-being can emerge and stabilise. Within the rest of the paper, I move to apply the vocabulary of symbiosis to an empirical study of care farming to explore the relations at play within multispecies therapeutic interventions. First though, I briefly introduce and explain the methodological practices which enabled and produced this study.

## Methods for multispecies STORYTELLING

This research is part of a larger research project exploring the relationships between health, place and animals.54 It draws on 55 semistructured interviews with representatives from community farms engaged in alternative agricultural paradigms, as well as with representatives from groups who visited these farms for therapeutic purposes. Research was conducted and informed by the UK Economic and Social Research Council’s six key principles of ethical research. All interviews were recorded, following the obtaining of informed consent from participants, and these were later transcribed. All participants have been assigned pseudonyms.

Semistructured interviews can create a space for people to tell stories and reflect on processes of becoming affected.55 In this way, interviews can reveal some of the intense, affective, emotional and embodied relationships between humans and animals, and the agency of more-than-human elements in the co-production of certain forms and affective states.56 These interviews sought to explore how animals’ roles, experiences and needs were conceptualised and understood by humans within the spaces of care farming, drawing on the dwelt and situated knowledge of the people who worked with, and encountered the farm’s livestock on a day-to-day basis. Interviews with individuals embedded in relationships with animals can shed light on affective practices and relationships at play within multispecies communities.57 Formal practices of patient and public involvement were not feasible or appropriate for this study.

Alongside interviews, ethnographic observation was carried out with the aim of tracing how the lives of humans and the lives of animals within the ‘common worlds’ of the farms were, to quote Pacini-Ketchabaw *et al*, ‘entangled, interconnected, mutually dependent, and therefore mutually ‘response-able’’ (p.151).58 This involved paying attention to the inchoate and processual life of the farm,59 providing a way of, what Lorimer calls, ‘bearing witness to life’s momentary acts and their multivariate expression’ (p.75),60 and exploring everyday lived human-animal relationships as they emerged.

Drawing on Curtis *et al*’s approaches to sampling and site selection for qualitative health research,61 I came to base my in-depth ethnographic observation at a small, community based, ‘alternative’, farm in Wales. Like many care farms, their desire to engage in offering therapeutic opportunities developed from the personal philosophies of the people running the enterprise. Viewing (and wanting to position) the farm space as a wider community resource, they developed relationships with a range of local organisations and invited groups to visit the farm roughly once a week. The groups that came to the farm tended to be heterogeneous. Dave, who worked for a local council scheme and visited the farm regularly with a group, described how, *‘we work with people at risk of substance abuse, criminal justice system, homelessness, and a range of different partners*’. Visitors got involved with feeding the animals, cleaning them out, moving the animals from field to field.

Following the conclusion of interviewing and observation, all transcripts and field notes were imported into NVivo for coding and analysis. Analysis took a ‘messy’ approach, acknowledging that ‘reality’ may be read in many different ways, not definite nor singular, consistent nor coherent—a way of keeping the world open.62 Following Milligan, ‘the process of analysis has not been viewed as developing a definitive account, rather it has been viewed as one means of trying to understand the inter-relations of multiple versions of reality, and in doing so, it serves to stress the interconnectivities between actants’ (p.109).63 The process was not an attempt to uncover some hidden truth within the data, but rather an attempt to identify recurrent themes and patterns of relations, exploring some of the multispecies stories of symbiotic togetherness, and what these stories and relationships might mean for different (human and non-human) bodies’ capacities to ‘affect and be affected’.64

In the rest of the article, I move to demonstrate how thinking symbiotically reveals different facets, narratives and experiences of multispecies therapeutic interventions; the ways in which these encounters might produce benefit to humans at the expense of animals (parasitic), produce benefit to humans in ways that do not impact on animals (commensal), or even—potentially—produce some form of benefit to humans and animals alike (mutualistic).

## STORIES OF BEING WELL TOGETHER

### Parasitic multispecies relations

Parasitism describes a symbiotic relationship in which one actant benefits and the other is harmed. It provides a valuable analytical lens through which to explore human-animal relations. Indeed, Bull argues that a focus on parasitism re-emphasises the politics of multispecies worlds,45 and that exploring parasitic relations provides a means of ‘engaging with the politics of multispecies codependencies’ (p.81).44 More specifically, Michel Serres’ figure of ‘the parasite’ proves a useful way of thinking through these relationships.65 Serres describes three coinciding ways relations can become parasitic. First, parasitism can involve ‘analysing’; intercepting relations and taking from another actant. Second, parasitism can involve ‘paralysing’; interrupting another actant’s ‘usual functioning’. Third, parasitism can involve ‘catalysing’; forcing other actants to act differently, in a way that they would not ordinarily.65–67 These subtypes of parasitic relations are not distinctive categories, but rather overlapping and entangled relationships, co-existent and interdependent.

In a parasitic means, humans on care farms come to depend on animal bodies to produce ‘vital flows’ of healthful relations. A focus on expanding human capacities through care farming can result in a converse reduction to the relations which animals’ bodies have. In the quest to realise an environment that has therapeutic potential for human visitors to the farm, animals often lose out, as relations become ‘tangentially redirected’ by parasitic practices.66 67

Animals are attempting to live their own animal lives.68 Their life practices are potentially in conflict with human conceptions and imaginations of ‘therapeutic spaces’. Animals are both complicit in, and importantly, resistant to, the various therapeutic practices and spaces in which they are enmeshed. I frequently observed animals keeping their distance, disrupting therapeutic ‘territorialisations’.64

Many care farms thus enact processes and practices that aim to make animals available for encounters, limiting animals’ mobilities and agency, and designing farm spaces to open up opportunities for interspecies relationships—paralysing and catalysing forms of parasitism. Developing opportunities for ‘therapeutic’ encounters with animals results in having to overcome particular animals’ desires not to be seen, deliberately making the animals more available and encounterable when otherwise they may seek isolation; reducing their opportunities to ‘speak back’ and inject their own agency into these relationships. Framing animals as always available further reifies a parasitic attitude towards animals’ positions in these spaces, with humans centred as recipients of therapeutic affect, and animals marginalised into objects.

Similarly, care farms often made moves to habituate their livestock, interrupting the animals' ‘usual functioning’,66 and forcing them to act differently, to present a specific imagination of a ‘therapeutic space’ and indeed, ‘animal-ness’.69 This frequently involved what Yarwood and Evans call a ‘sanitisation of livestock’, presenting clean and docile animals with ‘pet’ names,70 catalysing and paralysing animals to make them suitable for human contact. Humans have expectations of what animals should be like.68 The habituation of animals to human presence can come to be regarded as necessary and desirable in enabling human-animal relations that produce new human bodily capacities, framed by anthropocentrism and parasitism.

Encounters with animals may produce positive affective intensities for humans,8 9 but can be less conducive to animal flourishing and functioning. Farmers must balance care for animals with human curiosity, while creating and fulfilling the animal encounters for which people came to the farms. There is clearly the potential for conflict between harmonising both, and care farming practitioners, such as Dan and Diana struggled to manage these often conflicting practices:

As soon as we got to the farm, Dan was keen to show the visitors the new-born lambs, though he was also cautious, as when the lambs are little, he doesn’t want to bother them. It was interesting seeing his clear desire to show off the lambs and indulge the visitors, with his obvious concern about the sheep’s welfare. (Field notes)

Okay I think, maybe in the 8 years we've been going, at one point maybe one person stood on a chick and trampled it, and I’m very sorry about that chicken. (Diana, manager of a care farming programme)

In Diana’s story, the relation becomes not so much one of parasitism, but, given the potential for emotional distress arising from harming an animal,71 has the potential to become one of synnecrosis, a process in which both actants are harmed through the symbiotic relationship. Parasitism, in and of itself, can disrupt therapeutic processes when humans become aware of how these relationships and encounters are ‘rendered’ possible.72

The chick’s death also highlights that care farms operate, what Van Dooren describes as, a ‘regime of violent care’ (p.92); a process in which intimate care for some bodies and species sits alongside the domination, coercion and abandonment of others.73

We dip our feet all the time now, but our chickens were ill a lot more, coz obviously you've got more people going in there all the time. (Diana, manager of a care farming programme)

As well as more direct harms, the role of visitors to the farms acting as disease vectors and pathways must also be considered, as Diana indicates above. To quote Bigmore, ‘salmonella, coccidial oocysts and most of the major diseases can use the humble wellington boot as a form of transport’ (p.27).74 The increased human presence within the farms due to visiting groups seeking some form of therapeutic experience can lead to ill-health for the livestock that are being sought out for their therapeutic place-making associations.

The farms are not passive spaces, but require a level of work and labour to maintain their status, both as a farm, and as a place with a reputation for therapeutic experiences. There are tasks that must be completed to maintain the farm enterprise and uphold a level of care for the animals which are part of that enterprise. Focusing on assuring human well-being diverts time and labour from animal care:

It is a fine balance between making sure that the visitors are getting our utmost care and they always are a priority, but you also have priority of welfare of animals as well […] Yeah so there'll always be negative sides, and I think also, some days it is a real battle to get everything done, and we think actually, I wish I could have cleaned those chickens out better, or, I wish I could have given them a bit more food that day, but we always do the best we can and like I said, the key thing is always to reach the visitors. (Valerie, care farming project coordinator)

As Valerie describes, with often a finite amount of time to dedicate to this upkeep, the relations between humans and animals become framed and focused around a parasitic exchange that takes away care from the non-human. A focus on optimising the welfare of human participants can result in a converse reduction on animal welfare. The anthropocentric focus here produces a parasitising siphoning which diminishes the totality of relations and opportunities for the non-human actants to instead profit a proliferation of capabilities and capacities for humans. Human needs and desires become dominant over animals. Attempts are made to manage the relations and affects available in an ambition to allow for the place of the farm to act as a vector in affecting the human body’s power of acting49—in ways which parasitise non-human capabilities. Animals’ health and well-being can become neglected in the pursuit of fulfilling positive and healthful relations for the human visitors. Parasitism produces new bodily capacities by seizing and steering the relations available.67

These symbiotic relationships are incredibly complex and contingent, ambiguous and obfuscated, ever-shifting and changing. The directionality too can change. One day at the farm, a group was working to build windbreaks to help shelter the farm’s beehives. This was framed as an act of care, doing something that would help the bees. The visitors to the farm were excited and enthused, and felt good that they were doing something to benefit the bees. An encounter of potentially mutual benefit. Then someone got stung. To say that the humans are being parasitised here is perhaps a step too far, but it highlights the complex interplay within these encounters and relationships.

As Bull reminds us, multispecies living together is ‘a spectrum of parasitisms, commensualisms, mutualisms, predations, amensalisms and even synnecrosis’ (p.79).45 Such a spectrum is important in recognising complexity, and moving away from dichotomies of whether interspecies encounters are good or bad for (the health of) one or another species. With this in mind, I want to consider commensal relations, and what might be referred to as a level of ‘more-than-human indifference’75

### Commensal multispecies relations

Commensal relations are those in which one actant benefits from the relationship without causing either benefit or harm to the other actant involved in the relationship. Serres’ focus on parasitic relations being those based on an unequal exchange could include commensal relations; someone benefits, someone receives nothing—an unequal relationship.65 However, I argue that there is value in exploring commensal relations independently of parasitism. A focus purely on the uniformity of exchanges blurs the dynamics, failing to attend to the full spectrum of the transactional dynamics of living things and symbiotic livings together. There is a large difference between a human feeling some form of beneficial therapeutic affect because of an interaction which harms an animal, and a human feeling some form of therapeutic benefit from an interaction which causes neither benefit nor harm. Both are unequal exchanges, where something is ‘taken’ and nothing is ‘given’, what Serres may consider ‘analysing’ parasitism,66 however these are vastly different relationships.

Anthropological readings of commensal relations often describe the pathways through which certain species came to benefit from living alongside humans; dogs, cats and rats dwelling among and close to humans.76 77 Cassidy argues that commensals are those that are ‘clearly changed through living alongside human beings’ (p.10).78 There is a sense of human exceptionalism among such conceptualisations of commensalism though. Commensal relations do not change just animals, humans are also affected by living alongside other beings. Commensal relations are multidirectional, and humans can equally come to benefit from living alongside animals.

Haraway describes commensals as those who are neither benefactors nor parasites, but ‘devices with their own ends who/which hitch a ride’; ‘accompanying rather than companioning’, ‘more about “riding along with” rather than “cum panis”, that is, “eating bread with”’ (pp.234–254).79 Animals can influence the way relations with(in) place can unfold simply by being around, creating affective experiences.8 While some of the relationships between humans and animals on the farms often did specifically involve deliberate and practised forms of ‘encounter’, other animals were simply present within the wider farm environment.

The chickens, in their new run, further away from the main activity hub, appear to be separated and forgotten about by visitors. Despite being overlooked – people do occasionally remark that it is nice to have the animals around. Simply something that is present within the landscape. (Field notes)

Dan, one of the farmers interviewed, describes how ‘*there’s something really satisfying that makes me feel really well hearing the cock crowing down there and knowing there’s a bunch of chickens in the orchard’*. Animal presence can (re)shape the emotional geographies of place, triggering memories and sensuous affects. Here, the chickens are simply accompanying humans through the emergent ‘therapeutic space’ of the farm, rather than engaged in more parasitic relations that exploit animals’ bodies for a human proliferation of health. The groups that visited the farm for ‘therapeutic’ purposes have the opportunity to gain some form of benefit from the chickens’ presence that has little impact on the chickens themselves (the wider agricultural paradigm withstanding). A commensal relationship, based around opportunities for human benefit from the co-presence of animals, enabling human flourishing but offering little to the non-human actants that produce these relations. Though simultaneously, avoiding a parasitic symbiosis, producing therapeutic affect for humans without harming or hindering animals. Human bodily capacities are simply changed from being alongside animals, the sympoetic generation of a sense of well-being.15

Rich: What about the animals, do you think they get anything out of all the contact with people?

Joyce: Yeah I think the pigs do, I don’t think the chickens really care that much, but the pigs you know whenever we go up to the field for whatever reason, not just to look after them, but to go and do the harvesting or work on the vegetables, then yeah, they also get, they get a scratch behind the ears and things like, and something to eat perhaps some old cabbage leaves or something that kind of thing, yeah so they, I do think they get quite a bit of personal contact, and they definitely seem to appreciate that, even if there’s not food involved they seem to like, perhaps the friendship.

Lisa: The sheep, I don’t think they really care as long as they've got grass and they're not hassled, I don’t think they really care. Whereas the pigs, they like scratches and cuddles.

For Joyce and Lisa, both farmers facilitating visiting groups, the everyday relations between their chickens and sheep and the visiting humans is a much more commensal one. People enjoy their relationships and encounters with both the chickens and the sheep, affective engagements that produce and proliferate healthful capacities. However, the chickens and sheep are understood to be accompanying actants that co-constitute these possibilities. Not sharing in the affective benefits of these relations, yet not suffering because of them either. Here, animals become positioned more as tools to provoke some form of therapeutic encounter for humans, jettisoned as subjects of health in their own right. However, for both Joyce and Lisa, pigs are attributed a different relationship. The pigs are positioned as affective recipients of their relations with humans, appreciative of the interspecies sociality, viewed as partners in a relationship that multidirectionally distributes new bodily capacities. Drawing on this, I turn now to examining how ‘therapeutic’ relations between humans and animals in the spaces of care farms might instead be framed and practised in a more mutualistic manner.

### Mutualistic multispecies relations

Mutualism involves a symbiotic relationship in which both actants benefit from ‘living together’. In biological terms, it specifically refers to a relationship between different species (as opposed to co-operative relations, used to describe similar relations within a species). Harrison *et al* argue that geography has paid ‘insufficient attention to the nature and meaning of the mutualisms and adaptations that have evolved between the species’ particularly in applying understandings of mutualisms to ‘structures involving humans’ (p.436).80 And indeed, similar could be said for work within the medical humanities.

Hatch argues that the prevalent perspective surrounding therapeutic relations with animals is ‘what can animals do for us?’, with little consideration as to how such relations may affect animals.10 Hatch goes on to argue that there is a dearth of material that focuses on the possible ill effects of such ‘therapeutic’ relations on the animals themselves. I certainly agree with Hatch that considering the experiences of non-humans is important, and the previous sections of this paper have sought to contribute to the gaps they identify. However, I would also argue that focusing solely on the potential ill effects to animals and simply assuming that animals do not receive anything of benefit from these relationships at all is equally problematic. Indeed, as Haraway has argued, overly emphasising animal suffering tends to give rise to a view of animals as passive and lacking agency, simply receiving human action.79 There needs to be recognition of non-humans’ active participation in worldhood, understanding, as Hayward describes, ‘the ambivalent, powerful, and elusive ways’ that animals take part in interactions (p.186).33 A ‘therapeutic’ relationship with (an)other species does not always have to be asymmetrically and anthropocentrically parasitic or commensal; relations between humans and animals might instead result in a mutually beneficial proliferation of affective capacities.

However, a caveat is perhaps important here, particularly given the agricultural context of care farming. Indeed, it could be argued that care farming animals are simply being asked to provide an additional service, alongside and on top of their existing agriculturally productive roles; the logics of capitalism finding new surplus value to extract and render in the form of animal affect.72 Thus, it is worth acknowledging that the mutualisms expressed here are deeply (and perhaps, fundamentally) partial. Thinking about how these interspecies encounters can produce mutualistic benefits often requires, to quote Law, recognising that realities are ‘often (or always) vague, diffuse, uncertain, elusive and/or undecided’ (p.599);62 multispecies relationships are certainly not definite. While we should not close down the possibility that animals may benefit in certain ways from their relationships with humans within these ‘therapeutic’ spaces, equally, we must bear in mind the constraints within which these forms of flourishing operate and might be understood. The agricultural paradigm captures and precodes all of the interactions taking place within care farms, limiting and complicating the transactional symbiotic dynamics of living things.

Yet, here I am drawn to Hayward’s question, as to whether ‘there are modes of captivity here that do not rely on total domination as the only modality of power?’ (p.178).33 The limits imposed by the politics of domination here do not altogether preclude the possibilities for some (however small) reciprocal benefits. Indeed, the marginality of these mutualisms provides a platform from which to begin to reconsider these practices, for if there can be benefit to both human and animal alike, should we not work to enhance and amplify such possibilities? Again, following Hayward, ‘what is at play […] is more than a politics of domination’ (187).33 To ‘stay with the trouble’,15 involves recognising that problems raised by certain forms of human-animal relationships can often sit alongside powerful possibilities for non-human futures.73

So what additional benefits might animals accrue because of these relationships? Emel *et al* argue that certain forms of farming can work to enable, rather than overcome, animal agency. This ‘enabling’ creates the opportunity for what Emel *et al* describe as, ‘livelier livelihoods’, where humans and non-humans exist as counterparts in a socioecological system that produces viable and potentially enriching lives for all.81 The more regular contact with humans because of the additional human bodies on the farms has the potential to normalise livestock to human presence, in turn, constituting a less stressful experience for livestock during agricultural practices, as Diana and Valerie both explain:

I think also because we're with the animals all the time, they are more used to people being around, which means that sort of catching them for slaughter, 'oh look, there’s my friends, I’ll just get in this trailer', it makes it less stressful for them, collecting eggs from the chickens as well […] they're more used to us being in there, they're not frightened of us. (Valerie, care farming project coordinator)

I mean we're actually going in and feeding them every day so they're actually getting more used to bigger groups of people every day, so I think you probably can minimise the risk on, the negative impact on the animal. (Diana, manager of a care farming programme)

Here, although being involved in an agricultural system may not necessarily end well for animals, they do conceivably experience certain benefits from their relations with humans; the mutual entanglements enable non-humans to thrive and flourish, resisting and refracting territorialisations of a ‘health-denying’ place.82 Thus, while the additional human bodies on the farms can limit the relations available for non-humans as discussed earlier, this can also produce new potentials on the farms. Human-animal relations are situated and contingent, a consequence of particular relations between particular actants.

Having people visiting the farms for therapeutic purposes also creates a level of transparency and visibility for the livestock, forcing farmers to ensure that their animals are kept in better conditions, as Lisa describes:

We can't get anything with the sheep, if you know, you can't have a sheep die of maggots or you can’t have something lame for too long, so they do get a better care because they've got more people looking at them, and feeling responsible for them […] they get better care coz there’s more people looking at them.

Again, while having additional people visit the farms can produce parasitic relations (as discussed previously), these are only some of the sets of relations that can emerge in the ‘taking place’ of health.62 83 Instead of harming or hindering, opening up farm gates to visitors may instead serve to enrich the lives of the farm animals, providing new stimuli and affective relations.

I think welfare wise, one of the arguments that I think, for us doing this, is that we've got time to be able to do it, whereas I think the livestock team, they're often rushing to get things done, whereas we have the time to actually spend because these guys are paying to be supported here, we have time to be able to sit and watch sheep, and watch their behaviour and think, 'okay that one’s not right today' or 'why’s that not right'. (Valerie, care farming project coordinator)

As Valerie discusses, familiarity can create mutually beneficial affects for both humans and animals involved. An attachment and attunement to individual animals can produce new capacities to affect and be affected in humans, while also drawing new relations into an animal’s health assemblage. As Milligan describes, informal caregivers, like the care farm participants, ‘can offer crucial insights’ (p.326).84 Although the visitors are not professional agriculturalists, their ability to notice that an animal is ‘not right today’ as Valerie describes, due to the closer relationships formed with the livestock, can produce benefits for the animals. An element of reciprocity in care begins to emerge within the spaces of the care farms.

Greenhough and Roe,85 drawing on Acampora,86 develop the concept of a ‘somatic sensibility’, a compassionate concern for the ‘other’ as a proper object of ethical consideration, apprehended through the shared experiences of having a (vulnerable) body. Somatic sensibilities generate relations of ‘symphysis’, a state of growing together emergent through (inter)relationships of sharing.86 These concerns lead to becoming involved in animals’ lives, in multispecies emotional entanglements that lead not just to becoming therapeutic ‘with’,79 but becoming therapeutic ‘together’,73 producing new bodily capacities multidirectionally through mutualistic relations. It was often identifying the subjectivity of animals that allowed for the emergence of mutually transformative relationships between heterogeneous actants. As Hayward argues, seeing familiarity in other beings can offer empathy and critical engagement, ‘identification counters the objectification of radically different organisms’ (p.177).33 Doing the work of paying attention through the cultivation of this somatic sensibility elevates animals from ‘bare life’, to instead have ‘qualified lives’, biographical and political.87 88 Visitors to the farms frequently and actively invoked animals’ ‘biographies’ in this way to produce a (mutually) beneficial entanglement of multispecies stories.89 These entanglements acted to explicitly draw the care of humans and animals together, encouraging careful relationships for more-than-humans, reframing ideas about which bodies are important in, and should benefit from, these spaces:

We always say 'and this is Lilly, shes got one brown eye' - one of my group, he wouldn’t go anywhere near the stable - and they've got a story, so we say the story, so we say, 'Jamie do you wanna come in now and see the horse', 'Oh no, oh no, no I can’t wait to get out of here', and they're all like, ‘Oh no Jamie, come on, come and have a go, Lilly was an abandoned horse and she's only a couple of years, you know she was very small when we found her, she was very injured, and now we care for her, and she can't do you any harm at all Jamie, do you want to come on in?', 'Oh right', and you see him going in and you know he’s not making eye contact, then you see him touching the horse and then by the end, he's feeding the horse! Oh, it’s remarkable, it’s remarkable! (Alys, group leader)

As Plumwood describes, an encounter with someone else’s needs and reality creates ‘an interactive process in which each transforms and limits the other’ (p.156).90 There are links here to Haraway’s concept of ‘shared suffering’, that recognising animals as significant others produces consequential relationships. Haraway argues that ‘sharing pain promises disclosure, promises becoming’ (p.84).79 Engaging in the embodied experiences and histories of animals produces practices and ‘flows of becoming’,91 encouraging reciprocity and multidirectionally generating new capacities to affect and be affected. These narratives draw heterogeneous actants together, building what Tsing calls ‘a world of overlapping lifeways in which mutualistic transformation’ (p.258) might be possible.92

This is particularly visible in the work that several of the care farms did in rescuing horses who had been neglected or abandoned, rehabilitating and training them. The training was more about enabling and instilling ways for the horses’ everyday care to be manageable by humans, rather than for equestrian activities. This rehabilitation work specifically involved working with people affected by health conditions, as Alys explains:

We got to train the horses, so, the students would use a clicker and a treat reward system, and that was amazing. There was a boy with ADHD [attention deficit hyperactivity disorder], and we said, ‘you've got to really consider your behaviour, no sudden movements', so it was amazing to see him, having to really manage himself, which he did beautifully and then when he got a horse to do something, and then he rewarded it and clicked it, he was like 'wow', he saw, I guess he saw the benefits of realising his actions on others, and how his behaviour, if it’s altered, might have a positive effect on others, so that, for him, was massive. (Alys, group leader)

Here, the relationship between horse and human creates a productive line of flight that acts to retrain physical attributes and responses,93 an experience which, importantly, reshapes the bodily capacities of both human and animal to affect and be affected, defining new possibilities for horse and human alike. It is perhaps not a relationship of parity, but the relationship is one of mutualism. Reciprocity is important here, rather than a balanced equality. The equine participants receive a level of care, rehabilitation and opportunities for flourishing. However, this relationship also provides new ways of being for a variety of human groups. As Plumwood describes, relationships of mutuality allow us to ‘take joy in the flourishing of others’ (p.196).90

However, it is again worth bearing in mind the constraints within which these forms of ‘flourishing’ operate. Questions arise as to whether these horses want to become involved in relationships with humans. There are also considerations to be made around the possibilities for interspecies emotional contagion94 and more-than-human empathy.95 Yet, while such an existence may still result in the closing down of certain ways of being from how the animals are kept, it can equally be argued that it still results in the horses having a much greater power to act and the expansion of their capabilities than in their abandoned and neglected state; health is processual and relational. As Van Dooren reminds us, ‘the world beyond the fence is not Eden’ (p.114).73

Horse-human entanglements have affects on both species.93 As Argent notes, allowing for social agency in horses involves being open to the idea that horses can come to take pleasure from the synchronised corporeal behaviours and movements of human-horse interactions as much as humans.96 These encounters bring humans and horses together in practices that result in new capacities for both species, and can enable actants to move towards flourishing, even if only in the most incremental of steps. This could be about reducing an animal’s anxiety and fear of humans by gradually increasing its interactions with people, creating new possibilities. Rather than an existence shadowed by apprehension of people, these encounters may enable a horse to live a more healthful life; a sympoetic rehabilitation, making lives liveable again.15

Standing watching the horses, I thought back to the images I’d been shown from when they’d first arrived at the farm, neglected, unkempt, and mistreated, was a joy. Today, seeing them seeking attention, nuzzling, initiating contact, with the visitors (to the visitors delight), it’s hard not be cautiously optimistic that they may (slowly, warily) also ‘benefit’ in some ways from these visits. Allowing the animals agency, rather than forcing them to interact, seems key here. (Field notes)

The intentionality of these encounters is an important step in co-producing relationships which offer this potential for mutual flourishing; relationships which allow animals to ‘inject what might be termed their own agency into the scene’ (p.13).68 Spaces of care are ‘shared accomplishments’,97 though importantly here, this shared accomplishment is co-produced by more-than-human agencies. Humans and animals making each other ‘capable of something new in the world of multispecies relationships’ (p.19).15

Care farm enterprises can be seen to be aligning the provision (and, perhaps equally importantly, the funding) of care for humans and animals in ways that creates opportunities for both. A more-than-human therapeutic space emerges from these mutualistic relations, framed through a multidirectional and voluntary relationship that produces benefit to each being in their own right, rather than a means to an end.98 A space in which new relational and bodily possibilities for both human and non-human are possible as a result of this togetherness, where interspecies relations and associations produce a continuing vitality among different actants.92 Reciprocity is intimately interwoven in a co-production of care.99 Following Van Dooren, while these practices may be messy, flawed, imperfect, they represent opportunities to strive towards ways ‘to participate with care’ and grasp for more ethical modalities of relationship with non-human others.73

It’s about caring in the rural environment, for people, but also for the environment, and actually, we can give that little bit of extra care to the animals, that little bit of extra care to the environment, that little bit of extra care to potentially the hedges or the vegetables or whatever, just because of the care for people we do, and that’s what I like […] I think in one way I feel that probably a lot of our guys can give the animals more positive attention, if I think of sometimes the livestock team, how they would round up a load of chicks or whatever, I can assure you they do it much quicker than we do, but from the animal welfare point of view, I’m sure that we do a better job. (Diana, manager of a care farming programme)

While arguments can be made regarding the egalitarianism of the mutualisms discussed in this section, a focus on mutualism and other symbiotic relations provides a critical and dynamic way of understanding human-animal relations and the potential for more-than-human therapeutic spaces and practices. Reciprocity in caregiving can be immediate or delayed, physical or emotional;99 it is not about *equal* benefit, but *mutual* benefit. Indeed, as Haraway concludes, complete symmetry is not the point: ‘such relations are almost never symmetrical […] this is about living responsively’ (p74).79 Versions of mutuality are enacted differently across species.100

Ultimately, this is not about saying that these encounters fundamentally are therapeutic for the animals involved, but instead recognising ways of working and practising interspecies therapeutic interactions in ways that might provide opportunities for more-than-human benefit. Through doing so we might move somewhat to addressing Haraway’s questions of ‘how to become less deadly, more response-able, more attuned, more capable of surprise, more able to practice the arts of living and dying well in multispecies symbiosis, sympoiesis and symanimagenesis on a damaged planet’ (p.98).15 Indeed, in the context of policies of austerity and the state withdrawing from responsibilities of care, care farming perhaps represents what Van Dooren describes as ‘a practice of care that aims to nourish and sustain species and their living participants in far-from-ideal conditions, where the most desirable options simply are not available’ (p.116).73

## Conclusion

A more-than-human approach can trouble understandings of multispecies places, practices and relationships being understood as ‘therapeutic’; who are these therapeutic processes therapeutic for? My attention here has been to different and specific facets and relationships across these spectrums of multispecies living together, paying attention to, what Haraway calls, the ‘patterns and processes of situated and dynamic dilemmas and advantages’ (p.60).15

Such an approach demonstrates that attempting to perform and realise human imaginations of ‘therapeutic’ affects can rely on processes that interrupt animals’ functioning, a ‘parasitic’ subversion and refraction of the relations available to non-humans. Animals’ emergent ethical and ontological positions are often subjacent to human health concerns and considerations. Though equally in other cases, I demonstrated that therapeutic affect can emerge through more ‘commensal’ relations in which animals are simply present, ‘along for the ride’.79 New bodily capacities are produced for humans through a co-presence with animals, while animals remain unharmed and unhindered.

Indeed, animals are active and subjective partners within these multispecies relationships, capable of experiencing an expansion of bodily capacities as a result of human-animal relations in similar ways to humans. Relations between humans and animals can, at times, result in a ‘mutual’ proliferation of affective capacities, reciprocally beneficial (though perhaps not equally beneficial). Animals emerge as affective companions in healthful practices and ‘trans-species flows of becoming’.91 These human-animal entanglements can lead not just to becoming therapeutic ‘with’,79 but becoming therapeutic ‘together’.73

This article has provided new ways of thinking about and conceptualising multispecies engagements within a context of health and well-being. It demonstrates the opportunity for new multispecies politics, and a provocation to reconsider how care for humans and non-humans might be brought together, in ways that open up potentialities for mutual and more-than-human benefit. Exploring relations of symbiosis provides a useful way to frame and interrogate how interactions between heterogeneous actants differently define, enable and enact what different actants may become regarding their health, and the gaps, absences and negative relationships of care. These discussions have practical relevance for care farming and animal-assisted therapies, particularly in thinking about how these practices design and develop future encounters. Perhaps in ways that, to quote Van Dooren, ‘distribute the impacts of this relationship in such a way that humans take on more and more of the burden’ (p.106) to enable a better chance of flourishing among non-human actants.73

These discussions highlight that there are important opportunities to think more critically about how to practise interspecies relationships and practices in ways that are less parasitic, and instead framed more by attempts at producing opportunities for mutualistic flourishing. Healthful relations between species do not have to be limited to anthropocentric politics. Given that the capacity for animals beyond cats and dogs at traditional animal shelters is often limited, care farms could provide a crucial place in the world and mechanism for rescuing, rehabilitating and providing care for abandoned and neglected animals, aligning the provision and funding of care for humans and animals in ways that creates opportunities for both.

There are also implications here for the wider field of medical humanities, particularly given the nascent engagement and dialogue of the medical-health humanities with the more than human.101 Positioning humans at the centre and neglecting the roles of non-human actants within health and disease narratives results in unfinished stories. The medical humanities literature has done much work to recognise the experiences and perspectives of marginalised groups,6 might we not consider the marginality of animals, and how they become enrolled in systems and practices of health and care? Indeed, the medical humanities are often touted as offering a route that might increase the ‘empathy of physicians toward patients’ (p.2);101 perhaps the medical humanities has a role to play too in opening up the possibilities for more-than-human empathy.95

To draw on de Bary’s cautious language in his 1878 introduction of symbiosis, ‘by themselves, these phenomena may not seem to be important, and for some people it might have appeared unnecessary to pay attention to them’ (p.138).38 However, as I have shown here, considering symbiotic processes provides a useful framework through which to consider healthful practices and flows of becoming. As scholars within the medical humanities become more interested in, to quote Hanlon, the ‘ways in which bodies not only interact, but co-evolve with things’ (p.144),53 exploring relations of parasitism, commensalism and mutualism produces a valuable way to frame and interrogate the diversity of relations drawn together in the co-production of ‘therapeutic’ affects, spaces and relationships. Through doing so, we might avoid what Braidotti describes as an ‘opportunistic transspecies commodification of Life’ (p.60),91 and instead engage in more equitable framings of relationships between human and non-human, pursuing ways of ‘being well together’.

## Notes

1. John Berger (2009). *Why look at animals?* London: Penguin.

2. Sarah Atkinson et al. (2015). “‘The Medical’ and ‘Health’ in a Critical Medical Humanities.” *Journal of Medical Humanities*;36(1):71–81.

3. Richard Gorman (2017b). “Therapeutic landscapes and non-human animals: the roles and contested positions of animals within care farming assemblages.” *Social & Cultural Geography*;18(3):315–35.

4. Jamie Lorimer (2016). “Gut buddies: multispecies studies and the microbiome.” *Environmental Humanities*;8(1):57–76.

5. Lorraine Plourde (2014). “Cat Cafés, Affective Labor, and the Healing Boom in Japan.” *Japanese Studies*;34(2):115–33.

6. William Viney, Felicity Callard, and Angela Woods (2015). “Critical medical humanities: embracing entanglement, taking risks.” *Medical Humanities*;41(1):2–7.

7. Robert G. W. Kirk, Neil Pemberton, and Tom Quick (2019), ‘Being Well Together? Promoting Health and Well-Being through More than Human Collaboration and Companionship’, Medical Humanities 45, no. 1 (1 March 2019): 75–81.

8. Richard Gorman (2017c). “Thinking critically about health and human-animal relations: Therapeutic affect within spaces of care farming.” *Social Science & Medicine*.

9. Jan Hassink et al. (2017). “Exploring the Role of Farm Animals in Providing Care at Care Farms.” *Animals*;7(6):45.

10. Alison Hatch (2007). “The view from all fours: a look at an animal-assisted activity programme from the animals’ perspective.” *Anthrozoös*;20(1):37–50.

11. Sarah Whatmore (2006). “Materialist returns: practising cultural geography in and for a more-than-human world.” *Cultural Geographies*;13(4):600–9.

12. Tzachi Zamir (2006). “The moral basis of animal-assisted therapy.” *Society & Animals*;14(2):179–99.

13. Dorothea Iannuzzi and Andrew N Rowan (1991), “Ethical issues in animal-assisted therapy programme,” *Anthrozoös*.;4(3):154–63.

14. Jacques Derrida (2008). *The Animal That Therefore I Am*. New York: Fordham University Press.

15. Donna Jeanne Haraway (2016). *Staying with the trouble: making kin in the Chthulucene*. Durham: Duke University Press.

16. Nikki Evans and Clare Gray (2012). “The Practice and Ethics of Animal-Assisted Therapy with Children and Young People: Is It Enough that We Don’t Eat Our Co-Workers?” *Br J Soc Work*. 2012 Jun 1;42(4):600–17.

17. Rachel Hine, Jo Peacock, and Jules N. Pretty (2008). “Care Farming in the UK: Contexts, Benefits and Links with Therapeutic Communities.” *Therapeutic Communities*;29(3):245–60.

18. Care Farming UK (2017). *Care Farming in the UK and Ireland: Annual Survey 2016/17*.

19. R.T. Ferwerda-van Zonneveld, S.J. Oosting, and A. Kijlstra (2012). “Care farms as a short-break service for children with Autism Spectrum Disorders.” *NJAS-Wageningen Journal of Life Sciences*;59(1):35–40.

20. J. Hassink et al. (2007). “Current status and potential of care farms in the Netherlands.” *NJAS - Wageningen Journal of Life Sciences*. 2007 Jan 1;55(1):21–36.

21. Simone R. De Bruin et al. (2009). “Green care farms promote activity among elderly people with dementia.” *Journal of Housing for the Elderly*;23(4):368–89.

22. Chris Leck, Dominic Upton, and Nick Evans (2015). “Growing well‐beings: The positive experience of care farms.” *British journal of health psychology*;20(4):745–62.

23. M Elings and J Hassink (2008). “Green Care Farms, A Safe Community Between Illness or Addiction and the Wider Society.” *Therapeutic Communities*;29(3):245–60.

24. Sorana C. Iancu et al. (2014). “Mental health recovery on care farms and day centres: a qualitative comparative study of users’ perspectives.” *Disability and rehabilitation*;36(7):573–83.

25. Helen Elsey et al. (2018). “Assessing the impact of care farms on quality of life and offending: a pilot study among probation service users in England.” *BMJ Open*;8(3):1–11.

26. Peter Kraftl (2014). *Geographies of alternative education: Diverse learning spaces for children and young people.* Bristol: Policy Press.

27. Lina H. Ellingsen-Dalskau et al. (2015). “Autonomy support and need satisfaction in prevocational programme on care farms: The self-determination theory perspective.” *Work*;53(1):73–85.

28. Christine Milligan (2014). *Geographies of care.* In: Cockerham W, Dingwall R, Quah S, editors. *The Wiley-Blackwell Encyclopaedia of Health, Illness, Behaviour , and Society.* Chichester: Wiley-Blackwell.

29. Nick J. Fox (2013). “Creativity and health: An anti-humanist reflection.” *Health*;17(5):495–511.

30. Nick J. Fox and Katie J. Ward (2008). “What are health identities and how may we study them?” *Sociology of Health & Illness*;30(7):1007–21.

31. María Puig de la Bellacasa (2012). “‘Nothing comes without its world’: thinking with care.” *The Sociological Review*;60(2):197–216.

32. Steve Hinchliffe (2007). *Geographies of Nature: Societies, Environments, Ecologies.* London: SAGE Publications.

33. Eva Hayward (2012). “Sensational Jellyfish: Aquarium Affects and the Matter of Immersion.” *Differences*;23(3):161–96.

34. Alexandra Kaley, Chris Hatton, and Christine Milligan (2019). “Therapeutic spaces of care farming: Transformative or ameliorating?” *Social Science & Medicine*.

35. Richard Gorman (2017a). “Smelling Therapeutic Landscapes: Embodied Encounters within Spaces of Care Farming.” *Health & Place*;47:22–8.

36. Eva Hayward (2010). “FINGERYEYES: Impressions of Cup Corals.” *Cultural Anthropology*;25(4):577–99.

37. Bradford D. Martin and Ernest Schwab (2012). “Symbiosis: “Living Together” in Chaos.” *Studies in the History of Biology*;4(4):7–25.

38. Nathalie Oulhen, Barbara J Schulz, and Tyler J Carrier (2016). English translation of Heinrich Anton de Bary’s 1878 speech, “Die Erscheinung der Symbiose.” (“De la symbiose.”) *Symbiosis*;69(3):131–9.

39. Martin Jones (2009). “Phase space: geography, relational thinking, and beyond.” *Progress in Human Geography*;33(4):487–506.

40. Donna Jeanne Haraway (2003). *The companion species manifesto: Dogs, people, and significant otherness.* Prickly Paradigm Press Chicago.

41. Stefan Helmreich (2009). *Alien ocean: anthropological voyages in microbial seas.* Berkeley: University of California Press.

42. Stefan Helmreich (2011). “Homo microbis and the Figure of the Literal.” *Cultural Anthropology Online*;17:24.

43. Steve Hinchliffe (2011). *Co-evolution.* In: Gregory D, Johnston R, Pratt G, Watts M, Whatmore S, editors. *The Dictionary of Human Geography*. Malden, Mass: Blackwell.

44. Jacob Bull (2016). “Toxic skin and animal mops: ticks and humanimal vulnerabilities.” In: Käll LF, editor. *Bodies, Boundaries and Vulnerabilities.* Cham: Springer;81–98.

45. Jacob Bull (2014). “Between ticks and people: Responding to nearbys and contentments.” *Emotion, Space and Society*;12:73–84.

46. Gavin J. Andrews (2017). “‘Running hot’: Placing health in the life and course of the vital city.” *Social Science & Medicine*;175:209–14.

47. Beth Greenhough (2010). “Vitalist Geographies: Life and the More-Than-Human.” In: *Taking-place: Non-representational Theories and Geography*, edited by B, Anderson, and P, Harrison. Farnham: Ashgate.

48. Ian Buchanan (1997). “The problem of the body in Deleuze and Guattari, or, what can a body do?” *Body & Society*;3(3):73–91.

49. Cameron Duff (2010). “Towards a developmental ethology: Exploring Deleuze’s contribution to the study of health and human development.” *Health*;14(6):619–34.

50. Phil Emmerson (2017). “Thinking laughter beyond humour: Atmospheric refrains and ethical indeterminacies in spaces of care.” *Environment and Planning* A;49(9):2082–98.

51. Nick J. Fox (2002). “Refracting ‘health’: Deleuze, Guattari and body-self.” *Health*;6 (3):347–63.

52. Nick J. Fox (2011). “The ill-health assemblage: Beyond the body-with-organs.” *Health Sociology Review*;20(4):359–71.

53. Neil Hanlon (2014). “Doing health geography with feeling.” *Social Science & Medicine*;115:144–6.

54. Richard Gorman (2017), Health, Place, and Animals: The Co-Production of Therapeutic Geographies and Community Supported Agriculture Farms, Unpublished, Cardiff University.

55. Andrew Goffey and Lynne Pettinger (2014). “Refrains and assemblages: Exploring market negotiations and green subjectivity with Guattari.” *Subjectivity*;7(4):385–410.

56. Robyn Dowling, Kate Lloyd, and Sandra Suchet-Pearson (2017). “Qualitative methods II: ‘More-than-human’ methodologies and/in praxis.” *Progress in Human Geography*;41(6):823–31.

57. Catherine Johnston (2008). “Beyond the clearing: towards a dwelt animal geography.” *Progress in Human Geography*;32(5):633–49.

58. Veronica Pacini-Ketchabaw, Affrica Taylor, and Mindy Blaise (2016). “Decentring the human in multispecies ethnographies.” In: Taylor C, Hughes C, editors. *Posthuman Research Practices in Education*. New York: Palgrave Macmillan;149–67.

59. John-David Dewsbury (2003). “Witnessing space: ‘knowledge without contemplation’.” *Environment and Planning* A;35(11):1907–32.

60. Hayden Lorimer (2010). “Forces of Nature, Forms of Life: Calibrating Ethology and Phenomenology.” In: Harrison P, Anderson B, editors. *Taking-Place: Non-Representational Theories and Geography*. Farnham: Ashgate;55–78.

61. S Curtis et al. (2000). “Approaches to sampling and case selection in qualitative research: examples in the geography of health.” *Social Science & Medicine*;50(7):1001–14.

62. John Law (2007). “Making a Mess with Method.” In: Outhwaite W, Turner S, editors. *The SAGE Handbook of Social Science Methodology*. London: Sage;595–606.

63. Christine Milligan (2001). *Geographies of Care: Space, Place, and the Voluntary Sector*. Aldershot: Ashgate.

64. G. Deleuze and F. Guattari (2008). *A Thousand Plateaus*. London: Continuum.

65. M. Serres (2007). *The Parasite.* Minneapolis: University of Minnesota Press.

66. Steven D. Brown (2002). “SD Michel Serres: Science, translation and the logic of the parasite.” *Theory, Culture & Society*;19(3):1–27.

67. Thomas J. Puleo (2013). *Parasitizing landscape for UNESCO World Heritage.* Geoforum;45:337–45.

68. C. Philo and C. Wilbert (2000). *Animal Spaces, Beastly Places.* New York: Taylor & Francis.

69. Pyrs Gruffudd (2000). “Biological Cultivation: Lutbetkin’s Modernism at London Zoo in the 1930s.” In: Philo C, Wilbert C, editors. *Animal Spaces, Beastly Places*. London: Routledge;223–42.

70. Richard Yarwood and Nick Evans (2000). “Taking Stock of Farm Animals and Rurality.” In: Philo C, Wilbert C, editors. *Animal Spaces, Beastly Places*. London: Routledge;99–116.

71. Arnold Arluke (2006). *Just a dog: Understanding animal cruelty and ourselves*. Philadelphia: Temple University Press.

72. Nicole Shukin (2009). *Animal capital: rendering life in biopolitical times*. Minneapolis: University of Minnesota Press;306 p. (Posthumanities).

73. Thom Van Dooren (2014). *Flight ways: life and loss at the edge of extinction*. New York: Columbia University Press.

74. Jim Bigmore (2011). *An essential guide to footbaths and disinfectants.* International Poultry Production;19(4):27–9.

75. John Clayton (2019). “’Park Rats’; Exploring a Violent Continuum of More-than-Human Indifference and Post-Humanity’.” In Civic Space and Desire, edited by C Drozynski, and D Beljaars. Abingdon: Routledge.

76. Greger Larson and Dorian Q Fuller (2014). “The evolution of animal domestication.” *Annual Review of Ecology, Evolution, and Systematics*;45:115–36.

77. Melinda A. Zeder (2012). “The domestication of animals.” *Journal of Anthropological Research*;68(2):161–90.

78. R. Cassidy (2007). “Domestication Reconsidered.” In: Cassidy R, Mullin MH, editors. *Where the wild things are now: domestication reconsidered.* Oxford: Berg;1–26.

79. Donna Jeanne Haraway (2008). *When Species Meet.* Minneapolis: University of Minnesota Press.

80. Stephan Harrison et al. (2004). “Thinking across the divide: perspectives on the conversations between physical and human geography.” *Area*;36(4):435–42.

81. J. Emel, Connie Johnston, and Elisabeth Stoddard (2015). “Livelier livelihoods: Animal and Human Collaboration on the farm.” In: Gillespie K, Collard RC, editors. *Critical Animal Geographies: Politics, Intersections and Hierarchies in a Multispecies World*. London: Routledge;164–83.

82. Geoffrey DeVerteuil and Gavin J. Andrews (2007). “Surviving profoundly unhealthy places: the ambivalent, fragile and absent therapeutic landscapes of the Soviet Gulag.” In: Williams A, editor. *Therapeutic Landscapes.* Hampshire: Ashgate;273–90.

83. Gavin J. Andrews, Sandra Chen, and Samantha Myers (2014). “The ‘taking place’ of health and well-being: Towards non-representational theory.” *Social Science & Medicine*;108:210–22.

84. Christine Milligan (2006). “Caring for older people in the 21st century: ‘Notes from a small island’.” *Health & Place*;12(3):320–31.

85. Beth Greenhough and Emma Roe (2011). “Ethics, space, and somatic sensibilities: comparing relationships between scientific researchers and their human and animal experimental subjects.” *Environment and Planning D: Society and Space*;29(1):47–66.

86. R. Acampora (2006). *Corporal Compassion: Animal Ethics and Philosophy of Body.* Pittsburgh, PA: University of Pittsburgh Press.

87. G. Agamben (1998). *Homo Sacer: Sovereign Power and Bare Life.* Stanford University Press; 1998.

88. S Eben Kirksey and Stefan Helmreich (2010). “The emergence of multispecies ethnography.” *Cultural Anthropology*;25(4):545–76.

89. Thom Van Dooren and Deborah Bird Rose (2012). “Storied-places in a multispecies city.” *Humanimalia*;3(2):1–27.

90. V. Plumwood (2003). *Feminism and the Mastery of Nature.* New York: Taylor & Francis.

91. R. Braidotti (2013). *The Posthuman.* Oxford: Polity Press.

92. Anna Lowenhaupt Tsing (2015). *The mushroom at the end of the world: On the possibility of life in capitalist ruins.* Princeton: Princeton University Press.

93. Anita Maurstad, Dona Lee Davis, and Sarah Dean (2015). “What’s underfoot: Emplacing identity in practice among horse-human pairs.” In: Nyman J, Schuurman N, editors. *Affect, Space and Animals.* London: Routledge;107–20.

94. Min Hooi Yong and Ted Ruffman (2014). “Emotional contagion: Dogs and humans show a similar physiological response to human infant crying.” *Behavioural Processes*;108:155–65.

95. Megan Martha Donald (2018). “When care is defined by science: Exploring veterinary medicine through a more-than-human geography of empathy.” *Area*.

96. Gala Argent (2012). “Toward a privileging of the nonverbal: communication, corporeal synchrony and transcendence in humans and horses.” In: Smith JA, Mitchell RA, editors. *Experiencing animal minds An anthology of animal–human encounters.* New York: Columbia University Press;111–28.

97. David Conradson (2003). “Spaces of care in the city: the place of a community drop-in centre.” *Social & Cultural Geography*;4(4):507–25.

98. J. Tannenbaum (1989). *Veterinary Ethics.* Baltimore: Williams & Wilkins.

99. Christine Milligan and Christine Milligan (2016). “Place and informal care in an ageing society: reviewing the state of the art in geographical gerontology.” *Progress in Geography*;34(12):1558–76.

100. Annemarie Mol (2002). *The body multiple: Ontology in medical practice.* Durham: Duke University Press.

101. Sarah de Leeuw et al. (2018). “Geographies of Medical and Health Humanities: A Cross-Disciplinary Conversation.” *GeoHumanities*;4(2):285–334.
